# Supplementary material for: Glioblastoma Cells Counteract PARP Inhibition through Pro-Survival Induction of Lipid Droplets Synthesis and Utilization
Source: Cancers (Basel). 2022 Jan 30;14(3):726. doi: 10.3390/cancers14030726 (PMC8833394; doi:10.3390/cancers14030726)
Supplement: Supplementary file 1 [file cancers-14-00726-s001.zip › cancers-1483860-supplementary.pdf]

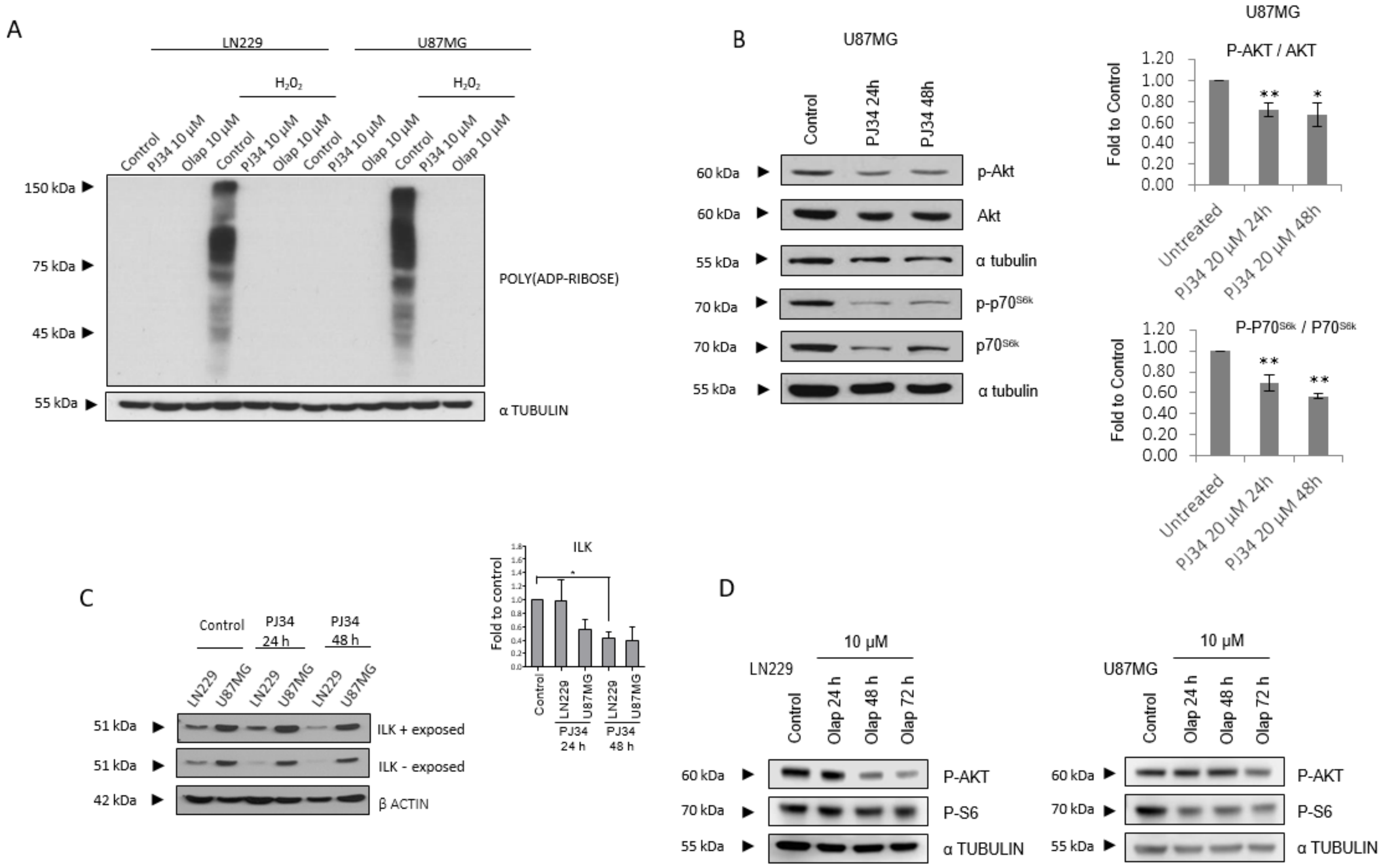

Figure S1. PARP inhibitors reduce poly(ADP-ribose) levels. PJ34 impaired the activation of AKT / mTORC1 pathway. Olaparib down-regulates mTOR pathway. (**A**) IB for poly(ADP-ribose) (PAR) (10H) (Millipore, MABC547) in LN229 and U87MG cells treated with PJ34 or Olaparib at the indicated dose for 90 min, in the presence or absence of H_2O_2. (**B**) IB for phospho-AKT and phospho-$P70^{S6k}$ in U87MG cells treated with PJ34 10 μM for 24 and 48 h. (**C**) IB for ILK in LN229 and U87MG cells treated with PJ34 10 μM for 24 and 48 hours. (**D**) IB for phospho-AKT and phospho-S6 in LN229 and U87MG cells treated with Olaparib 10 μM for 24, 48 and 72 h. Data are representative of 3 independent experiments. Relative abundance of a specific protein (Bands) based in two methods for normalizing WBs: (i) housekeeping protein normalization α-tubulin or β-actin (ii) total protein normalization, total Akt, $P70^{S6k}$. Values are mean ± SEM. * $p < 0.05$, ** $p < 0.01$, *** $p < 0.001$. Student's t test.

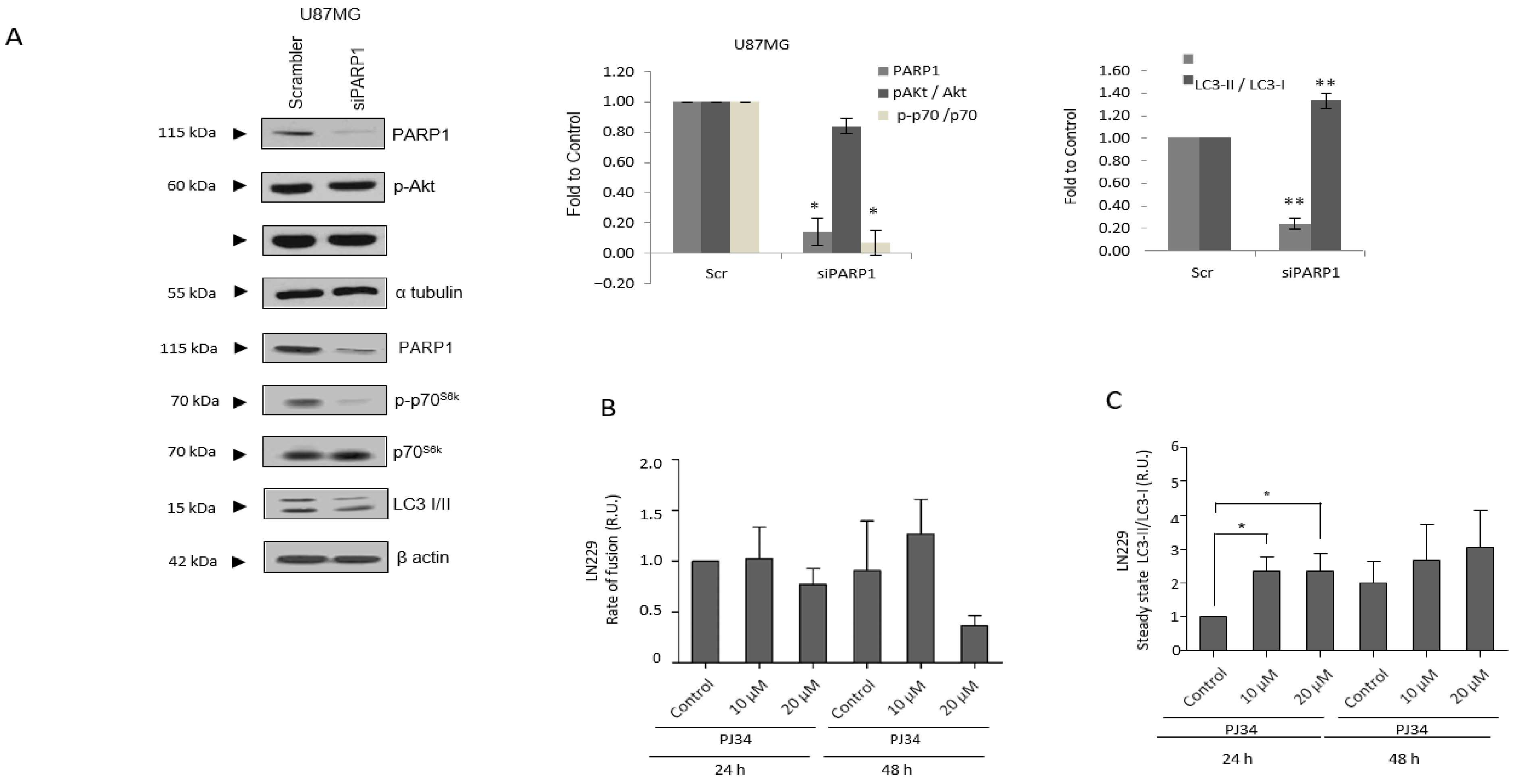

Figure S2. Effect of PARP1 silencing in PTEN mutant cells U87MG on AKT/mTORC1 / autophagy axis. PJ34 modulates autophagy in LN229 CELLS. (**A**) IB and graphical representation for the indicated protein in U87MG cells treated with PJ34 at the indicated doses and times. (**B**) Graphical representation of steady state LC3-II/LC3-I, in LN229 treated with PJ34 at the indicated doses and times, in the presence or absence of Lys Inh for 4 h. (**C**) Graphical representation of the autophagic rate of fusion, in LN229 treated with PJ34 at the indicated doses and times, in the presence or absence of Lys Inh for 4 h. Data are representative of 6 independent experiments (autophagy assays) and 3 independent experiments (western blots). Relative abundance of a specific protein (Bands) based in two methods for normalizing WBs: (i) housekeeping protein normalization α-tubulin or β-actin (ii) total protein normalization, total Akt, $P70^{S6k}$ Values are mean ± SEM. * $p < 0.05$, ** $p < 0.01$, *** $p < 0.001$. Student's t test.

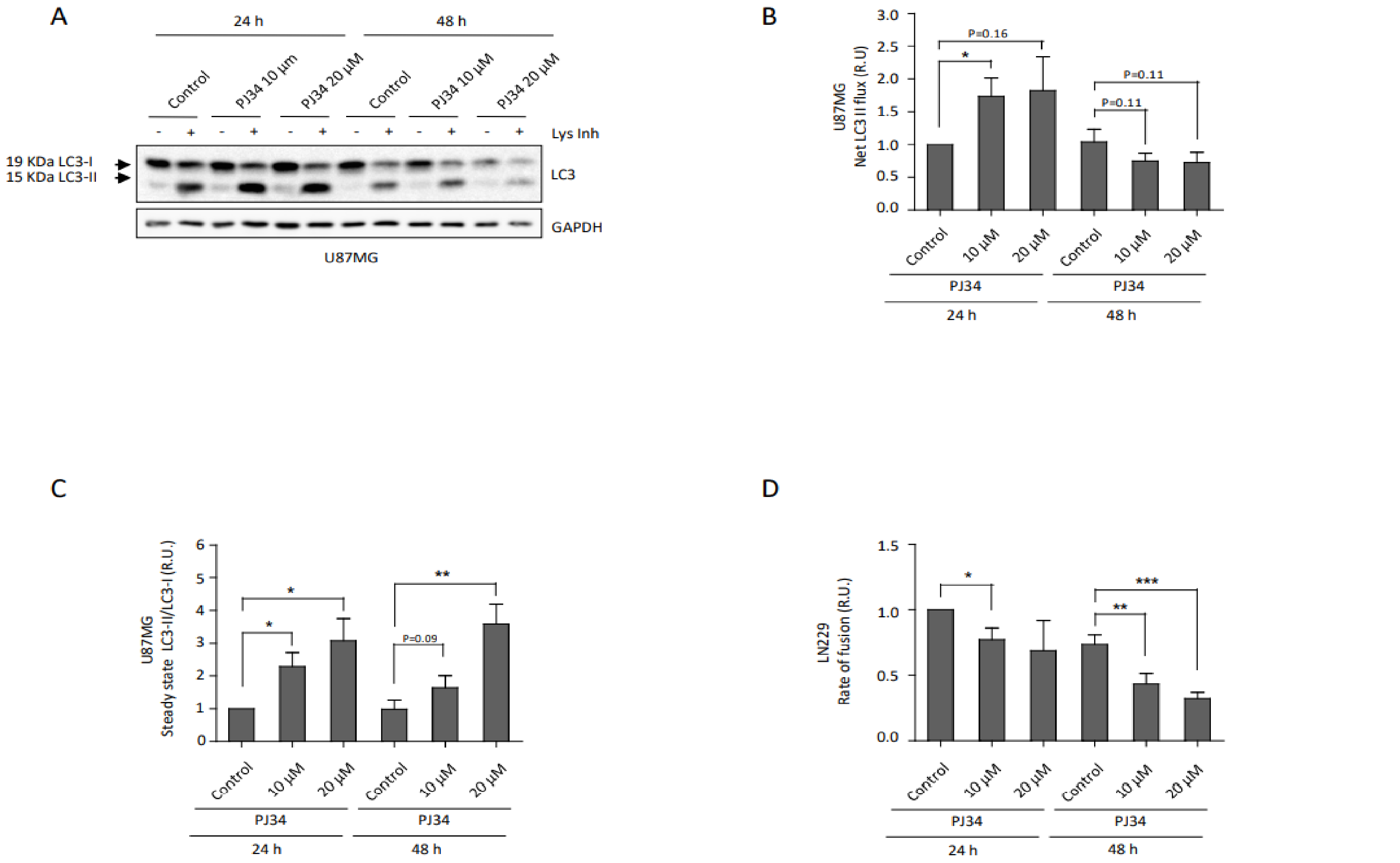

Figure S3. PJ34 modulates autophagy in U87MG cells. (**A**) IB for LC3 in U87MG cells treated with PJ34 at the indicated doses and times, in the presence or absence of lysosomal inhibitors (Lys Inh) for 4 h. (**B**) Graphical representation of net autophagic flux. (**C**) Graphical representation of steady state LC3-II/LC3-I. (**D**) Graphical representation of the autophagic rate of fusion. Data are representative of 6 independent experiments. Relative abundance of a specific LC3-II Band to LC3-I band were determined and normalized to housekeeping protein GAPDH. Values are mean ± SEM. * $p < 0.05$, ** $p < 0.01$, *** $p < 0.001$. Student's *t* test.

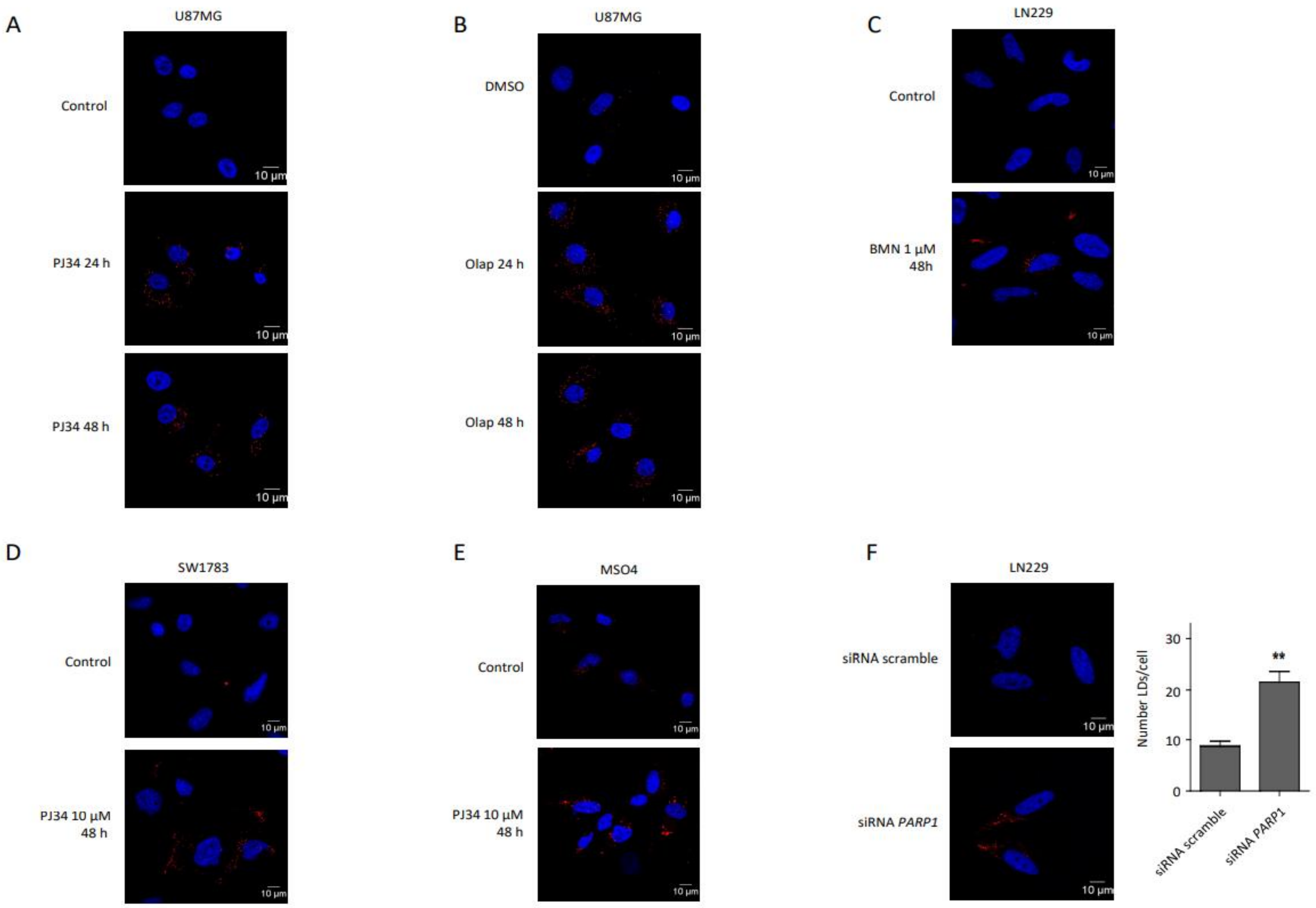

Figure S4. PARP inhibition induces lipid droplets formation. (**A**) Sudan red staining in U87MG cells treated with PJ34 10 µM at the indicated times. (**B**) Sudan red staining in U87MG cells treated with Olaparib 10 µM at the indicated times. (**C**) Sudan red staining in LN229 cells treated with BMN at the indicated dose and time. (**D**) Sudan red staining in SW1783 cells treated with PJ34 at the indicated dose and time. (**E**) Sudan red staining in MSO4 cells treated with PJ34 at the indicated dose and time. (**F**) Sudan red staining in LN229 cells transfected with scramble siRNA or siRNA against *PARP-1*. Lipid droplets quantitation is shown in right panel. Data are representative of 6 independent experiments. Values are mean ± SEM. * $p < 0.05$, ** $p < 0.01$, *** $p < 0.001$. Student's t test.

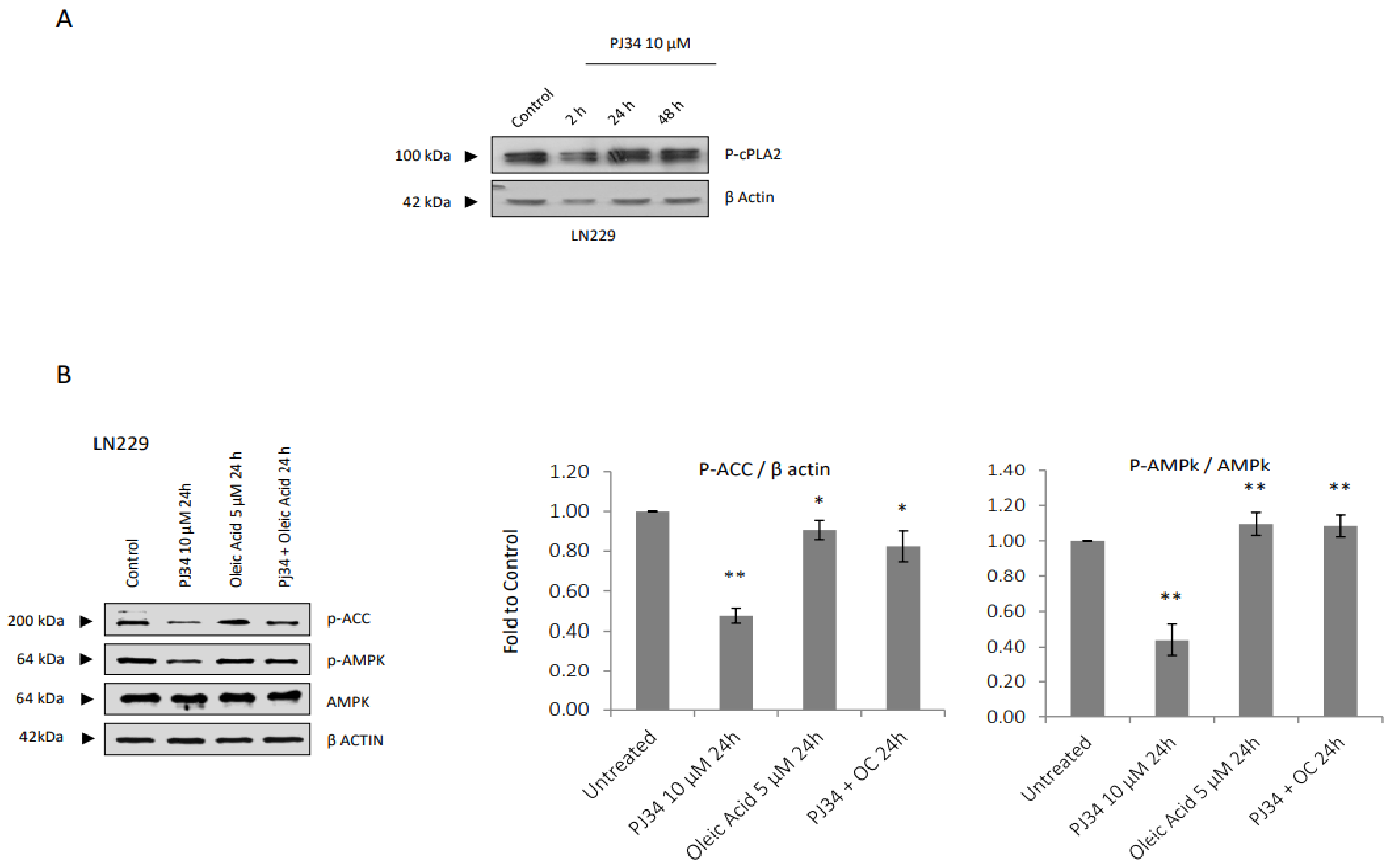

Figure S5. Phospho cPLA2 levels are not modulated by PARP inhibition. OA didn't compromise the ACC/AMPK axis in glioblastoma cells. (**A**) IB for phospho cPLA2 in LN229 cells treated with PJ34 at the indicated dose and times. (**B**) IB and graphical representation for the indicated proteins in LN229 cells treated with PJ34 and Oleic Acid (OA) at the indicated doses and times. Data are representative of 3 independent experiments. Relative abundance of each band was quantified and normalized to (i) housekeeping protein normalization β-actin (ii) total protein normalization, total AMPK. Values are mean ± SEM. * $p < 0.05$, ** $p < 0.01$. Student's t test.

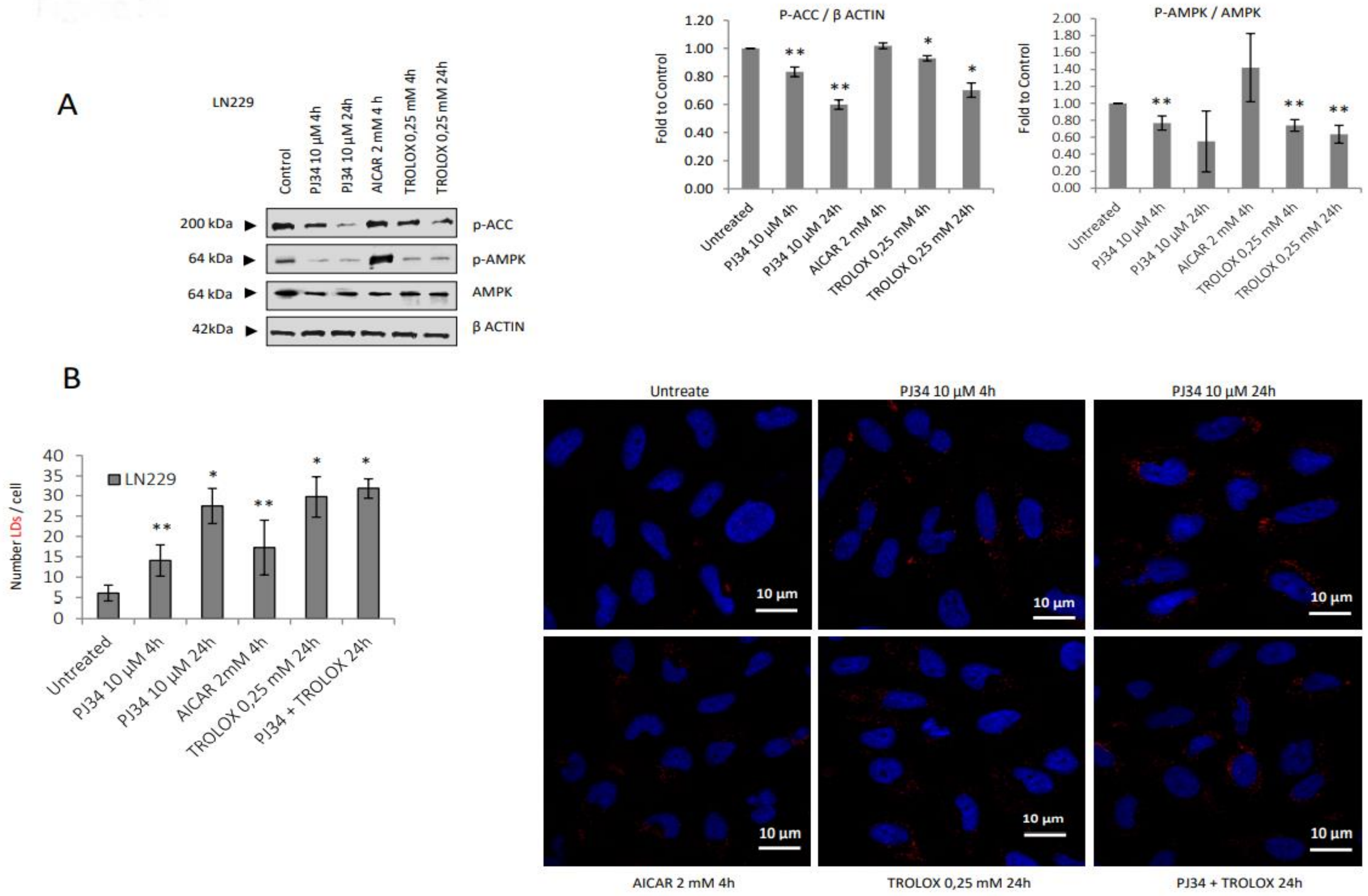

Figure S6. Antioxidant TROLOX downregulates ACC/AMPK pathway promoting LDs accumulation. (**A**) IB and graphical representation for P-ACC and P-AMPK in LN229 treated with PJ34 10 μM, AICAR 2 mM and TROLOX 0.25 mM for 4 and 24 h. (**B**) Sudan red staining in LN229 cells treated with PJ34 10 μM, AICAR 2 mM and TROLOX 0.25 mM for 4 and 24 h. Lipid droplets quantification is shown in left panel. Data are representative of 3 independent experiments. Relative abundance of each band was quantified and normalized to (i) housekeeping protein normalization β-actin (ii) total protein normalization, total AMPK. Values are mean ± SEM. * $p < 0.05$, ** $p < 0.01$, *** $p < 0.001$. Student's t test.

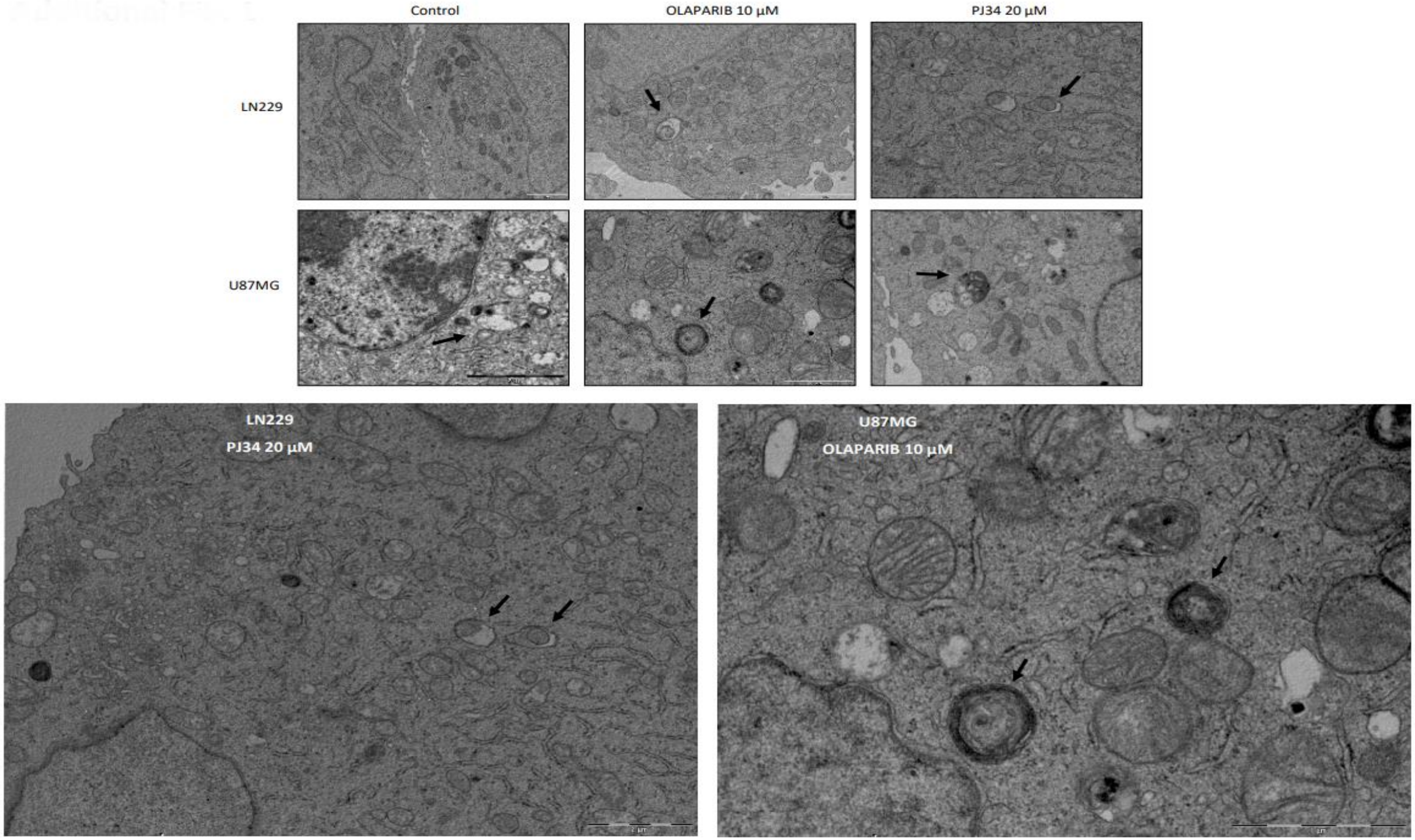

Figure S7. Representative electron micrographs depicting autophagosomes (indicated by black arrows) in LN229 and U87MG treated with Olaparib or PJ34 at the indicated doses for 48 h. Magnifications.

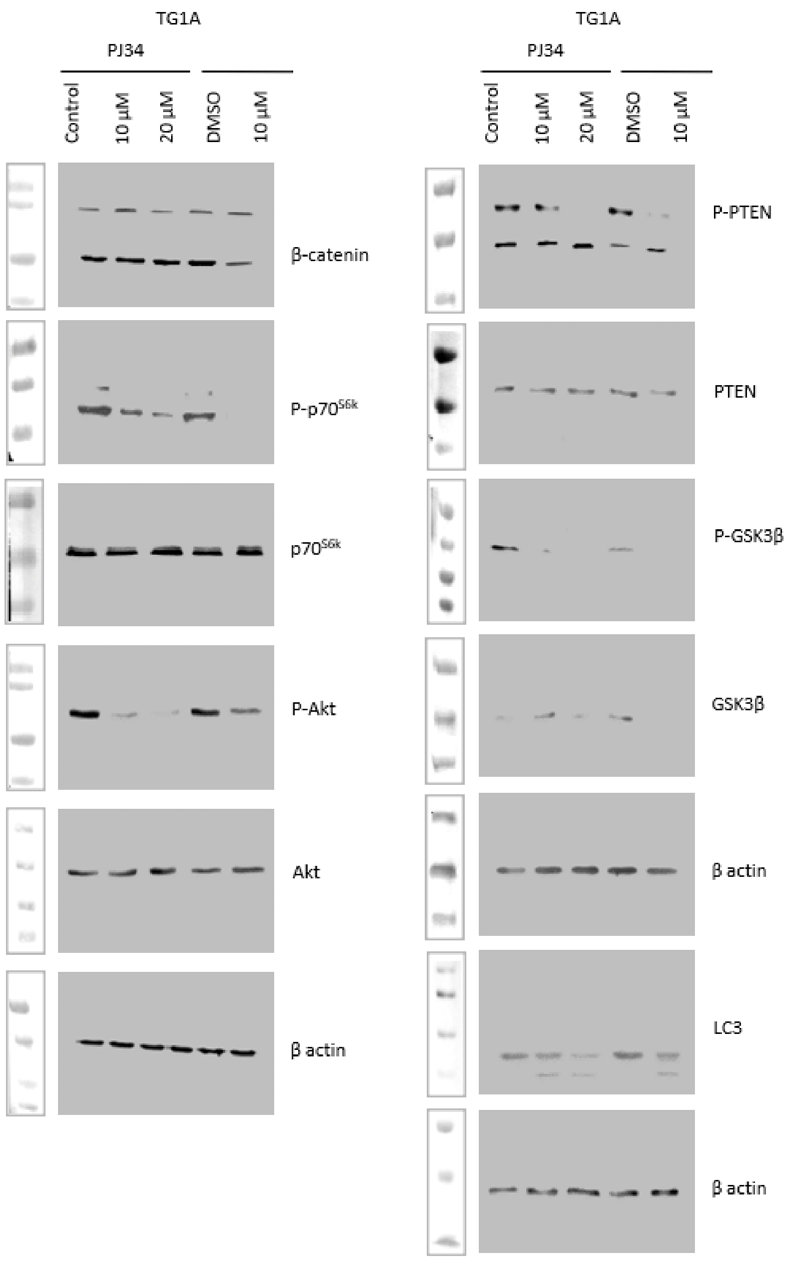

TG1A
PJ34
Control
10 µM
20 µM
DMSO
10 µM
β-catenin
P-p70S6k
p70S6k
P-Akt
Akt
β actin
TG1A
PJ34
Control
10 µM
20 µM
DMSO
10 µM
P-PTEN
PTEN
P-GSK3β
GSK3β
β actin
LC3
β actin

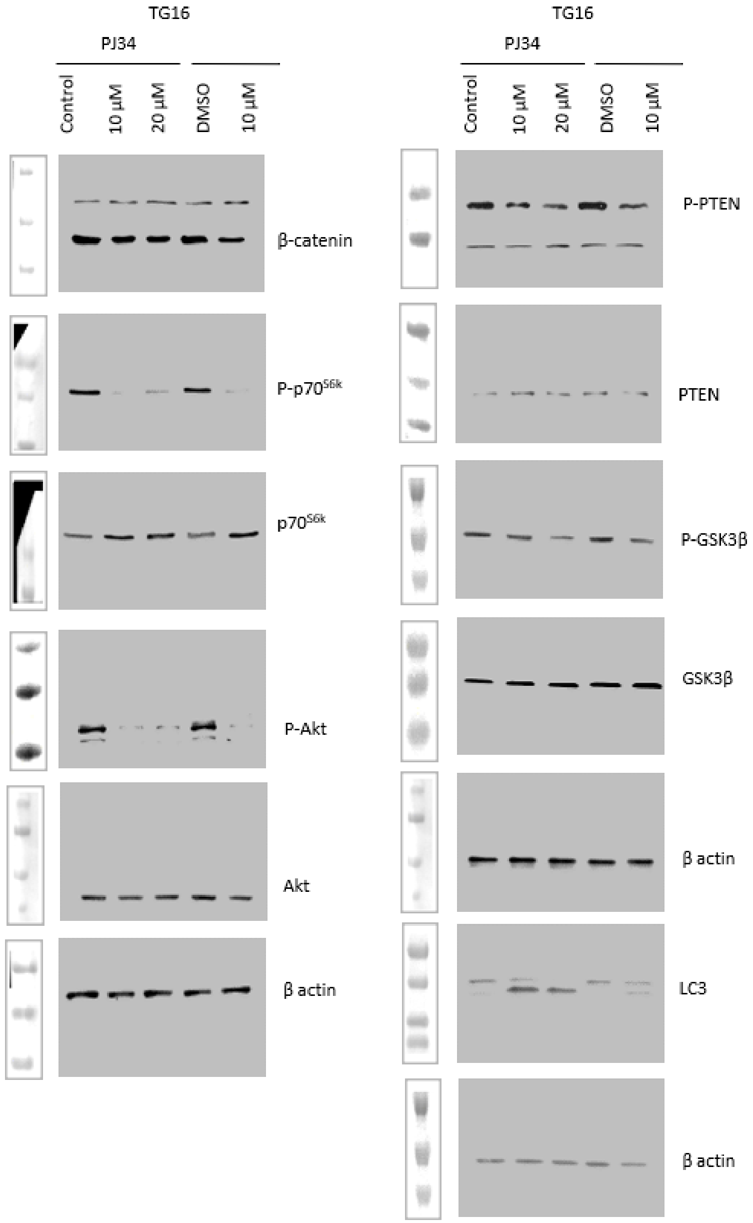
TG16
PJ34
Control
10 μM
20 μM
DMSO
10 μM
β-catenin
P-p70S6k
p70S6k
P-Akt
Akt
β actin
TG16
PJ34
Control
10 μM
20 μM
DMSO
10 μM
P-PTEN
PTEN
P-GSK3β
GSK3β
β actin
LC3
β actin

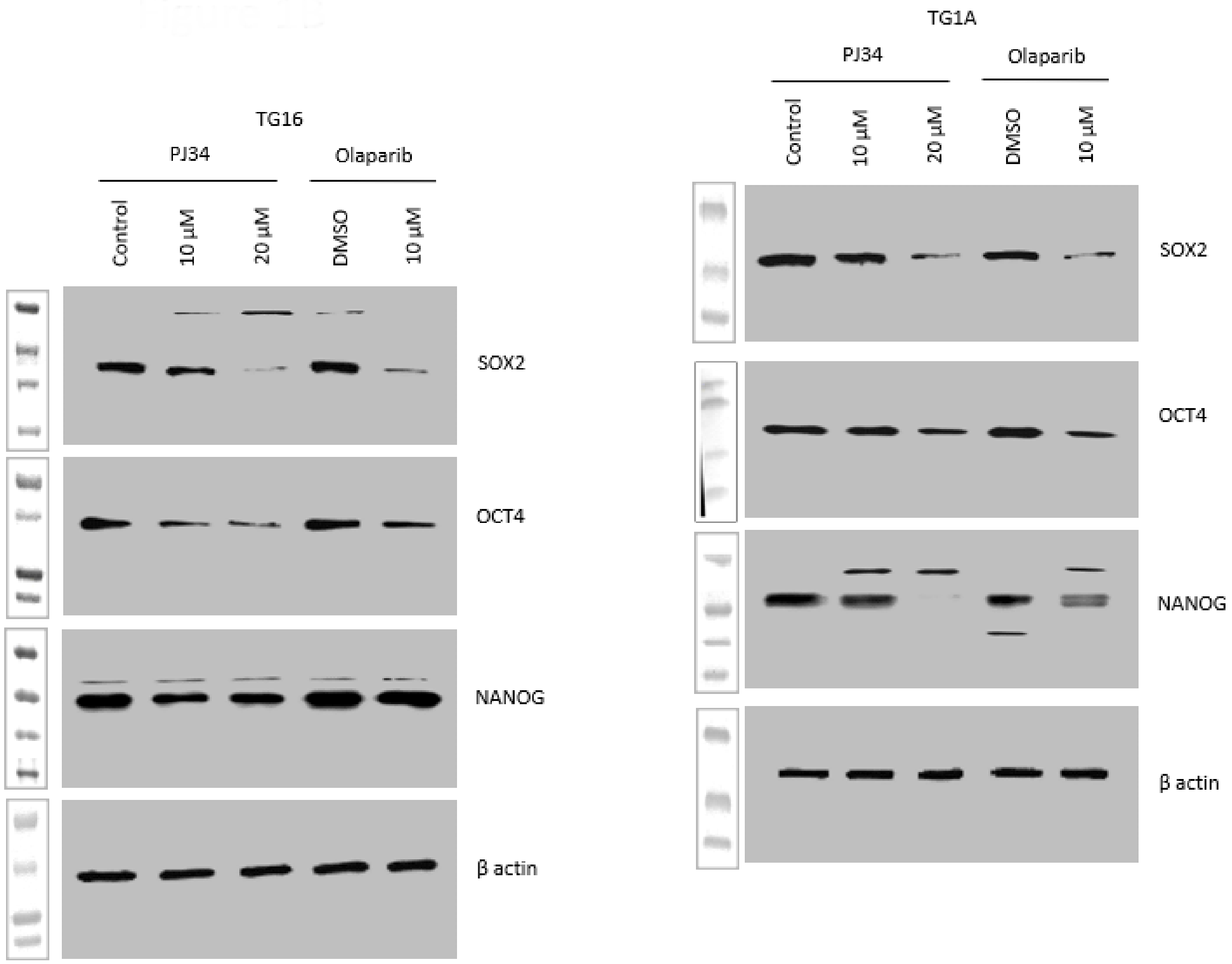

Figure S8. Uncropped Western Blots of Figure 1A,B.

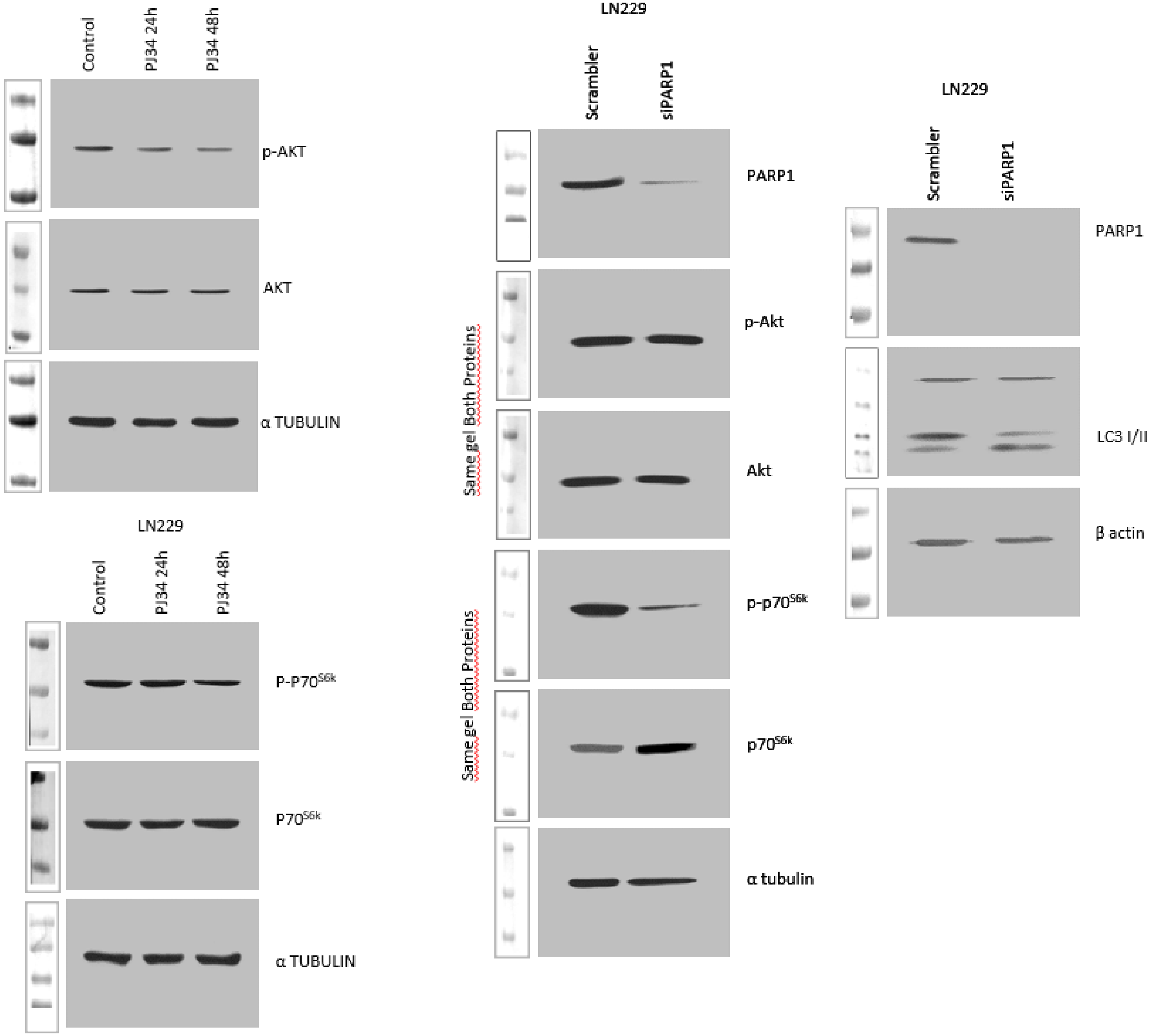

Control
PJ34 24h
PJ34 48h
p-AKT
AKT
α TUBULIN
LN229
Control
PJ34 24h
PJ34 48h
P-P70S6k
P70S6k
α TUBULIN
LN229
Scrambler
siPARP1
PARP1
p-Akt
Akt
p-p70S6k
p70S6k
α tubulin
Same gel Both Proteins
Same gel Both Proteins
LN229
Scrambler
siPARP1
PARP1
LC3 I/II
β actin

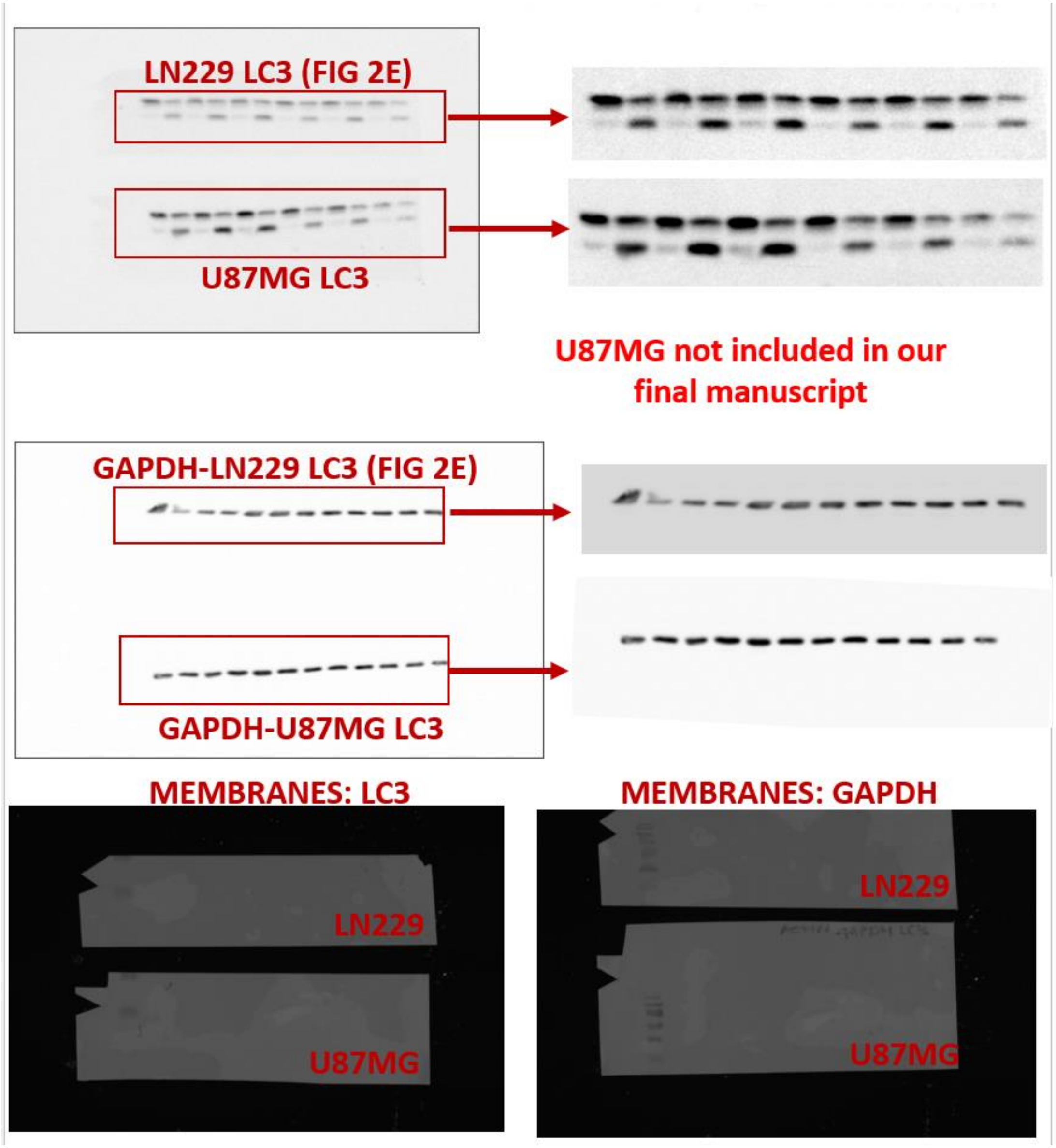

Figure S9. Uncropped Western Blots of Figure 2A,B,E.

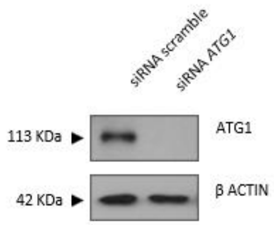

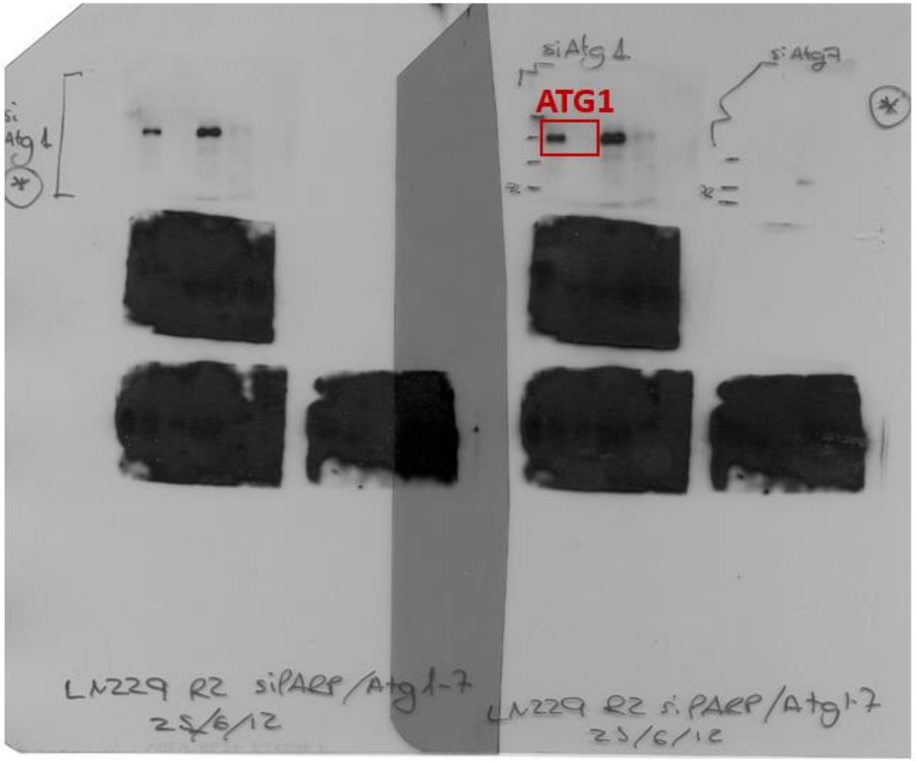

Figure S10. Uncropped Western Blots of Figure 3B.

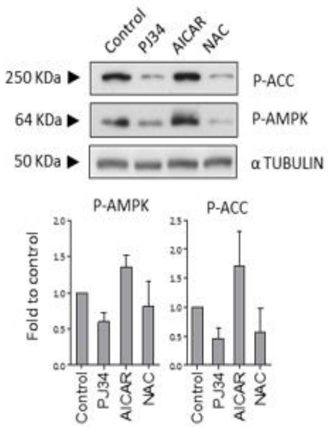
Control
PJ34
AICAR
NAC
250 KDa ▶
P-ACC
64 KDa ▶
P-AMPK
50 KDa ▶
α TUBULIN
P-AMPK
P-ACC
Fold to control
Control
PJ34
AICAR
NAC
Control
PJ34
AICAR
NAC

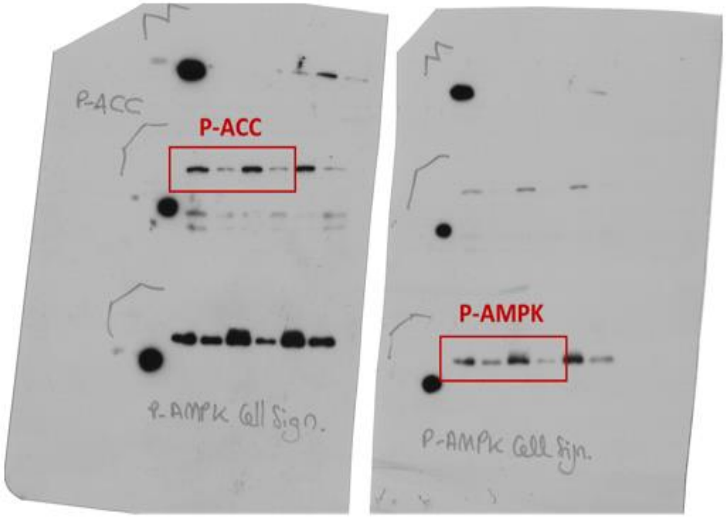
P-ACC
P-ACC
P-AMPK
P-AMPK Cell Sign.
P-AMPK Cell Sign.

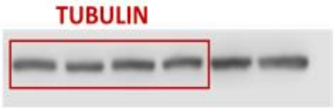
TUBULIN

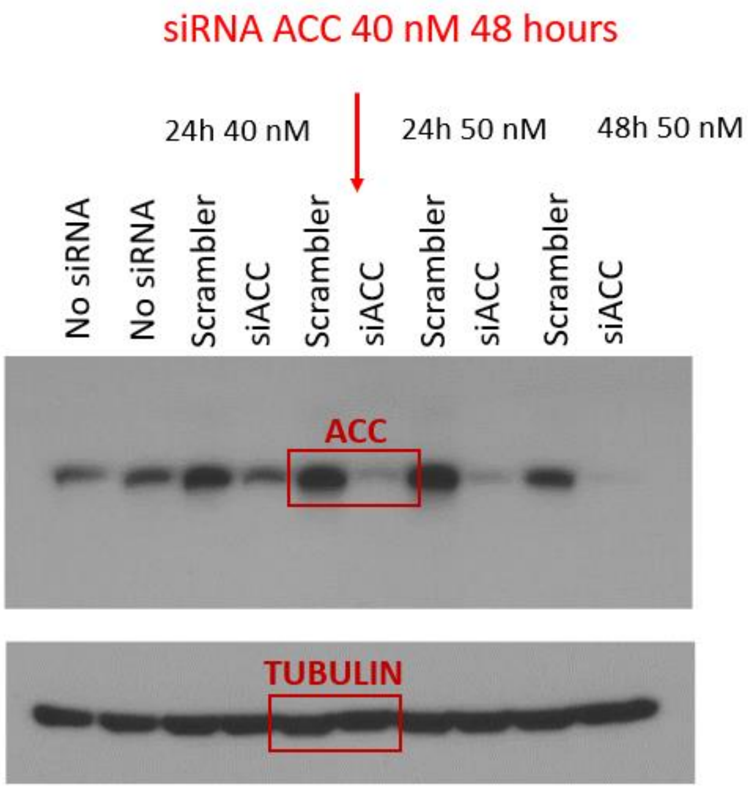

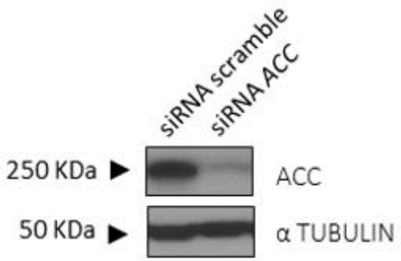

Figure S11. Uncropped Western Blots of Figure 4B,E.
